# Diagnosis and Treatment of Prostate Adenocarcinoma

**DOI:** 10.3390/cancers13153660

**Published:** 2021-07-21

**Authors:** Shoji Kimura, Takahiro Kimura

**Affiliations:** Department of Urology, The Jikei University School of Medicine, Tokyo 105-8461, Japan

Prostate cancer is the second most common cancer and the fifth leading cause of cancer-related death among men worldwide [1]. In recent years, significant advancements in the diagnostic and therapeutic modalities for prostate cancer have revolutionized its management in daily practice. New modalities such as robot-assisted radical prostatectomy and intensity-modulated radiation therapy have been widely used worldwide. However, the detection and management of recurrent tumors remain unresolved problems. Genetic medicine has been introduced for the diagnosis and treatment of prostate cancer. In this Special Issue, experts in this field reviewed the recent progress in important topics associated with prostate cancer. We present two systematic reviews and eight original articles on this topic. In this editorial, we summarize the main findings of the articles included in the Special Issue.

Chang et al. [2] reported the difference in oncologic outcomes among prostatectomy modalities, such as open, laparoscopic, and robotic surgeries, using the National Cancer Database in Taiwan. They found no significant differences in the positive surgical margin and biochemical recurrence rate among patients who underwent open, laparoscopic, or robot-assisted radical prostatectomy after propensity score adjustment. They further examined the effect of hospital volume on positive surgical margin and biochemical recurrence-free survival rates in patients with prostate cancer undergoing robot-assisted or non-robot-assisted radical prostatectomy [3]. They found that hospital volume significantly affected positive surgical margin rates and biochemical recurrence-free survival rates and concluded that robot-assisted radical prostatectomy should be performed in a relatively high-volume hospital (>100 patients/year), if patients with prostate cancer want to receive it.

Wu et al. [4] evaluated the monetary cost of the number of urological clinic consults, hospitalization rate and the cost for surgical complications in patients with prostate cancer who underwent open, laparoscopic, or robot-assisted radical prostatectomy. They found that medical resource consumption in the robot-assisted radical prostatectomy group was less than that in the open and laparoscopic groups because of its shorter learning curve.

Wu et al. [5] compared the oncologic outcomes of radical prostatectomy to those of intensity-modulated radiation therapy and androgen deprivation therapy in relatively young patients with intermediate prostate cancer. They found that radical prostatectomy was better than intensity-modulated radiation therapy plus androgen deprivation therapy in terms of oncologic outcomes in these patients.

Choi et al. [6] evaluated the oncologic outcomes of external beam radiotherapy in localized prostate cancer and compared various risk classification tools such as NCCN, D’Amico, AUA and CPG. They analyzed 1573 patients treated with external beam radiotherapy and classified them using a risk stratification tool with the highest predictive power for biochemical recurrence-free survival. They found that the NCCN risk classification had the highest predictive power. They concluded that dose escalation with modern high-precision techniques may increase survival in the high-risk group, but not in the low-risk group, although conclusive results of prospective studies using the NCCN risk classification are being anticipated.

Milonas et al. [7] assessed the risk of cancer-specific mortality and other-cause mortality of 1921 patients post-radical prostatectomy using the postoperative International Society of Urological Pathology Grade Group (GG) model. They found that the GG model showed high and consistent performance (time-dependent AUC: 0.88) in predicting cancer-specific mortality. 

Kimura et al. [8] performed a systematic review and meta-analysis of the impact of PSA persistence on oncologic outcomes after radical prostatectomy. They found that PSA persistence 4–8 weeks after radical prostatectomy was associated with biochemical and disease recurrence as well as cancer-specific mortality. Furthermore, they found that PSA persistence after radical prostatectomy was associated with disease recurrence in a subgroup of patients with pathologic nodal involvement.

Abufaraj et al. [9] performed a systematic review of the functional outcomes after local salvage therapies for patients with radiation-recurrent prostate cancer. They found that the rates of severe incontinence and erectile dysfunction were high after local salvage therapies, such as radical prostatectomy, HIFU, cryotherapy and brachytherapy for radiation-recurrent disease. They concluded that, despite these adverse consequences, the oncologic advantage may justify the use of local salvage therapy after radiation failure in select informed patients who had undergone a balanced and shared decision-making process.

Nguyen-Dumont et al. [10] performed multigene panel resting and evaluated the risk of aggressive prostate cancer in a case-control study. They found that males who carry *BRCA1*, *BRCA2,* and *ATM* germline pathogenic variants are at an increased risk of aggressive disease. They concluded that these rare genetic variants could be incorporated into risk prediction models to improve their precision in identifying males at a higher risk of aggressive prostate cancer and those with newly diagnosed prostate cancer who require urgent treatment.

Ando et al. [11] reported the impact of a high-sensitivity modified Glasgow prognostic score in patients with castration-resistant prostate cancer who received docetaxel. Using multivariate analysis, they found that a high-sensitivity modified Glasgow prognostic score ≥ 1 was a significant poor prognostic factor for overall survival.

In conclusion, this Special Issue provides updated information on the prognostic factors for non-metastatic prostate cancer after curative therapy, the functional outcomes of local therapy for radiation-recurrent disease, the impact of rare genetic variants on aggressive prostate cancer, and prognostic factors in patients with castration-resistant prostate cancer.
